# Allelic effects on starch structure and properties of six starch biosynthetic genes in a rice recombinant inbred line population

**DOI:** 10.1186/s12284-015-0046-5

**Published:** 2015-03-04

**Authors:** Jixun Luo, Stephen A Jobling, Anthony Millar, Matthew K Morell, Zhongyi Li

**Affiliations:** CSIRO Agriculture Flagship, GPO Box 1600, Canberra, ACT 2601 Australia; College of Medicine, Biology and Environment, Australian National University, Canberra, ACT 0200 Australia; International Rice Research Institute, Maligaya, Muñoz, Nueva Ecija Philippines

**Keywords:** Rice, Starch, Starch synthases, Starch branching enzymes, Starch property, RVA, DSC

## Abstract

**Background:**

The genetic diversity of six starch biosynthetic genes (*Wx*, *SSI*, *SSIIa*, *SBEI*, *SBEIIa* and *SBEIIb*) in *indica* and *japonica* rices opens an opportunity to produce a new variety with more favourable grain starch quality. However, there is limited information about the effects of these six gene allele combinations on starch structure and properties. A recombinant inbred line population from a cross between *indica* and *japonica* varieties offers opportunities to combine specific alleles of the six genes.

**Results:**

The allelic (*indica* vs *japonica*) effects of six starch biosynthetic genes on starch structure, functional properties, and abundance of granule bound proteins in rice grains were investigated in a common genetic background using a recombinant inbred line population. The *indica Wx* (*Wxi*) allele played a major role while *indica SSI* (*SSIi*), *japonica SSIIa* (*SSIIaj*) and *indica SBEI* (*SBEIi*) alleles had minor roles on the increase of amylose content. *SSIIaj* and *japonica SBEIIb* (*SBEIIbj*) alleles had a major and a minor role on high ratio of ∑DP ≤ 10 to ∑DP ≤ 24 fractions (R_CL10/24_), respectively. Both major alleles (*Wxi* and *SSIIaj*) reduced peak viscosity (PV), onset, peak and end gelatinization temperatures (GTs) of amylopectin, and increased amylose-lipid complex dissociation enthalpy compared with their counterpart-alleles, respectively. *SBEIIai* and *SBEIIbj* decreased PV, whereas *SSIi* and *SBEIIbj* decreased FV. *SBEIi* reduced setback viscosity and gelatinization enthalpy. R_CL10/24_ of chain length distribution in amylopectin is negatively correlated with PV and BD of paste property and GTs of thermal properties. We also report RILs with superior starch properties combining *Wxi*, *SSIj*, *SSIIaj*, *SBEIi* and *SBEIIbj* alleles. Additionally, a clear relation is drawn to starch biosynthetic gene alleles, starch structure, properties, and abundance of granule bound starch biosynthetic enzymes inside starch granules.

**Conclusions:**

Rice *Wxi* and *SSIIaj* alleles play major roles, while *SSIi*, *SBEIi*, *SBEIIai* and *SBEIIbj* alleles have minor roles in the determination of starch properties between *indica* and *japonica* rice through starch structural modification. The combination of these alleles is a key factor for starch quality improvement in rice breeding programs. R_CL10/24_ value is critical for starch structure and property determination.

## Background

Rice is the most important cereal crop in developing countries. It feeds over half of the world’s population, which is critical to sustain population growth (Toriyama et al. 2005). In rice grains starch is the major component that primarily controls rice quality (Umemoto et al. 2008). There are two major rice subspecies, *indica* and *japonica*. The diversity of starch properties between *indica* and *japonica* varieties have been widely studied, and the alleles of genes that affect the traits have been found through a number of approaches (Han et al. 2004; Tian et al. 2009, 2010; Kharabian-Masouleh et al. 2011, 2012; Zhao et al. 2011). In terms of rice traits, cooking and sensory properties are the major determinants for consumers’ acceptance of a rice variety. In the absence of a clear knowledge of sensory properties, amylose content, gelatinization temperature and gel consistency of rice flour have been widely used as important indicators of quality for rice varietal development (Cuevas and Fitzgerald 2012).

Amylose content (AC) of rice starch, affecting the cooking and eating properties, is determined by the activity of granule-bound starch synthase I (GBSSI) encoded by the *Wx* gene (Wang et al. 1995; Umemoto and Terashima 2002). A number of alleles were reported to be associated with starch AC variation. However, *Wx*^*a*^ and *Wx*^*b*^ dominate the functional alleles among *indica* and *japonica* subspecies, with high and intermediate GBSSI protein production, respectively (Sano 1984; Wang et al. 1995). They can be distinguished mainly by a G/T single nucleotide polymorphism (SNP) at 5′ splicing site of the first intron (Hirano et al. 1998) that resulted in differential splicing of the first intron. Gel consistency in rice is also related to AC (Tan & Corke 2002; Septiningsih et al. 2003; Zheng et al. 2007) and its quantitative trait locus mapped to the *Wx* locus has been reported (He et al. 1999; Lanceras et al. 2000).

Gelatinization temperature of rice starch is mainly determined by starch synthase IIa (SSIIa) (Govindaraj et al. 2009; Cuevas et al. 2010; Gao et al. 2011). Allelic effects of *SSIIa* in rice were reported earlier, and up to 9 SNP alleles were identified (Nakamura et al. 2005b; Umemoto and Aoki 2005; Bao et al. 2006; Yu et al. 2010). Two replacements of amino acid residues at the C-terminal region of the SSIIa protein were found to affect SSIIa activity and amylopectin structure and properties in grains (Nakamura et al. 2005b).

Aside from *Wx* and *SSIIa*, other starch synthetic genes are also involved in the modification of amylopectin structure and starch properties in rice varieties. The expression of *SSI* gene in *indica* rice is lower than that of *japonica* rice which reduces the synthesis of short chains in *indica* rice amylopectin (Takemoto-Kuno et al. 2006). A mutation in the *SBEI* gene leads to a decrease in long chains (DP ≥ 37) and intermediate chains (DP 12 to 21), increases in short chains (DP ≤ 10) and intermediate chains (DP 24 to 34) and a reduction in the onset, peak and end gelatinization temperature (Xie et al. 1999; Satoh et al. 2003). For the *SBEIIb* gene, a SNP was determined by Han et al. (2004) in the 3′UTR, between *indica* and *japonica* varieties of rice. Taking advantages of nucleotide polymorphisms, genetic markers have been developed for genotyping a series of starch biosynthetic genes to improve grain starch quality in rice breeding programs (Han et al. 2004; Yan et al. 2007; Tian et al. 2010). Nonetheless, nearly all these studies are carried out in isolated individual breeding lines with diverse genetic backgrounds.

The starch properties are highly related to the composition and chemical structure of starch not only in rice but also other cereal grains. Two types of glucan polysaccharides, amylose and amylopectin, comprise starch. The former molecule (approximately 20 ~ 30% of starch) is a long linear glucose polymer (~1% branched points) which is synthesised by GBSSI, through adding ADP-glucose to existing α-(1–4) glucan chains (Nelson and Rines 1962; Tsai 1974; Fedoroff et al. 1983; Shure et al. 1983). The latter (approximately 70 ~ 80% of starch) is a much larger molecule with frequent α-(1–6) branches formed by multiple enzymes, for example, starch synthases (SSs) and starch branching enzymes (SBEs) (Tetlow et al. 2004; Hannah and James 2008). The isoforms of SSs and SBEs differ in their activities in amylopectin synthesis in a developing endosperm. SSI elongates very short glucan chains to a degree of polymerisation (DP) 10 (Commuri and Keeling 2001; Fujita et al. 2006), while SSIIa is endosperm specific and is involved in the polymerization of intermediate chains (DP 10 ~ 24) by elongating short chains (DP < 10) of amylopectin (Nakamura et al. 2005b). The formation of α-(1–6) linkage in amylopectin molecules is mainly performed by SBEI and SBEII (Gao et al. 1996). SBEI transfers glucan chains with a wide range of DP ≤ 35, while SBEIIa is involved in the transferring of chains with DP 6 ~ 15 and SBEIIb forms almost only short chains of DP 6 and 7 (Mizuno et al. 2001; Nishi et al. 2001; Nakamura et al. 2010). The activities of these enzymes potentially affect starch structure and properties.

In this study, the polymorphic effects of six starch biosynthetic genes (*Wx*, *SSI*, *SSIIa*, *SBEI*, *SBEIIa* and *SBEIIb*) on starch structure and properties were studied in a common genetic background, using a recombinant inbred line (RIL) population derived from crossing IR64 (*indica*, *i*) and Nipponbare (*japonica*, *j*). With two alleles (*i* or *j*) for six different genes there are 64 potential combinations theoretically. In this work we have compared *i* or *j* alleles for each gene in a common background, with 12 combinations representing 64 as a whole. Different approaches such as amylose content assay using size-exclusion chromatography, chain length determination by capillary electrophoresis (CE), rapid viscosity analysis (RVA), and differential scanning calorimetry (DSC) were performed to analyse the starch structure and properties of these samples. Starch granule bound proteins (GBPs) were analysed to study the relation between those proteins and starch structure and properties.

## Results

### Grouping of recombinant inbred lines

**Figure 1:**
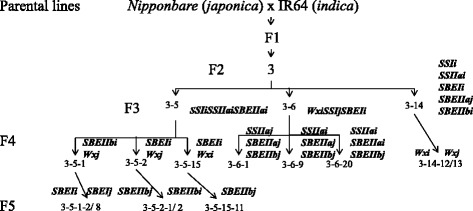
**Generation of RILs for each of two alleles of six starch synthetic genes.** Six genes for genotyping of alleles are *Wx*, *SSI*, *SSIIa*, *SBEI*, *SBEIIa*, *SBEIIb*. Parental lines are IR64 and Nipponbare. The arrows indicate descendants of each line. The numbers with slash line indicate the name of each line. The numbers following ‘F’ imply the generation on the left of the figure. The italic letters followed by ‘*i*’ or ‘*j*’ (indicating *indica* and *japonica*, respectively) indicate alleles of the RILs.

Six allele groups were selected from recombinant inbred lines corresponding to the 6 major starch synthetic enzyme genes, and parental lines (IR64 and Nipponbare) differing in their alleles (*i* vs *j*) for each of the 6 genes. Thus we segregated the RILs into six allele groups according to the allele combinations (Figure 1) to analyse the effects of *i* vs *j* alleles of different starch biosynthetic genes on starch structure and properties. The allele types of these six genes of each RIL are described in Table 1, and are designated as follows: line 3-14-12 and 3-14-13 as *Wxi* and *Wxj*; line 3-5-15-11 and 3-6-20 as *SSIi* and *SSIj*; line 3-6-9 and 3-6-1 as *SSIIai* and *SSIIaj*; line 3-5-1-2 and 3-5-1-8 as *SBEIi* and *SBEIj*; line 3-6-20 and 3-6-9 as *SBEIIai* and *SBEIIaj;* and line 3-5-2-2 and 3-5-2-1 as *SBEIIbi* and *SBEIIbj*. Meanwhile, we also separated RILs in *Wxi* and *Wxj* allele groups in some of the following analyses, because *Wx* alleles were well known in determining various starch characteristics.

**Table 1:** **Genotypes of RILs of six starch biosynthetic enzyme groups**

**Genotype**	***Wxi***	***Wxj***	***SSIi***	***SSIj***	***SSIIai***	***SSIIaj***	***SBEIi***	***SBEIj***	***SBEIIai***	***SBEIIaj***	***SBEIIbi***	***SBEIIbj***
**Lines**	3-14-12	3-14-13	3-5-15-11	3-6-20	3-6-9	3-6-1	3-5-1-2	3-5-1-8	3-6-20	3-6-9	3-5-2-2	3-5-2-1
***Wx***	i	j	i		i		j		i		j	
***SSI***	i		i	j	j		i		j		i	
***SSIIa***	i		i		i	j	i		i		i	
***SBEI***	i		i		i		i	j	i		i	
***SBEIIa***	j		i		j		i		i	j	i	
***SBEIIb***	i		j		j		i		j		i	j

Names of each RIL are shown in the first row of corresponding column. ‘i' and ‘j’ are short abbreviation for *indica* and *japonica* genotypes, indicating their parental origins, respectively. The second row of each column indicates the corresponding RILs. The names of six starch biosynthetic genes are listed in the first column.

### Amylose content

**Figure 2:**
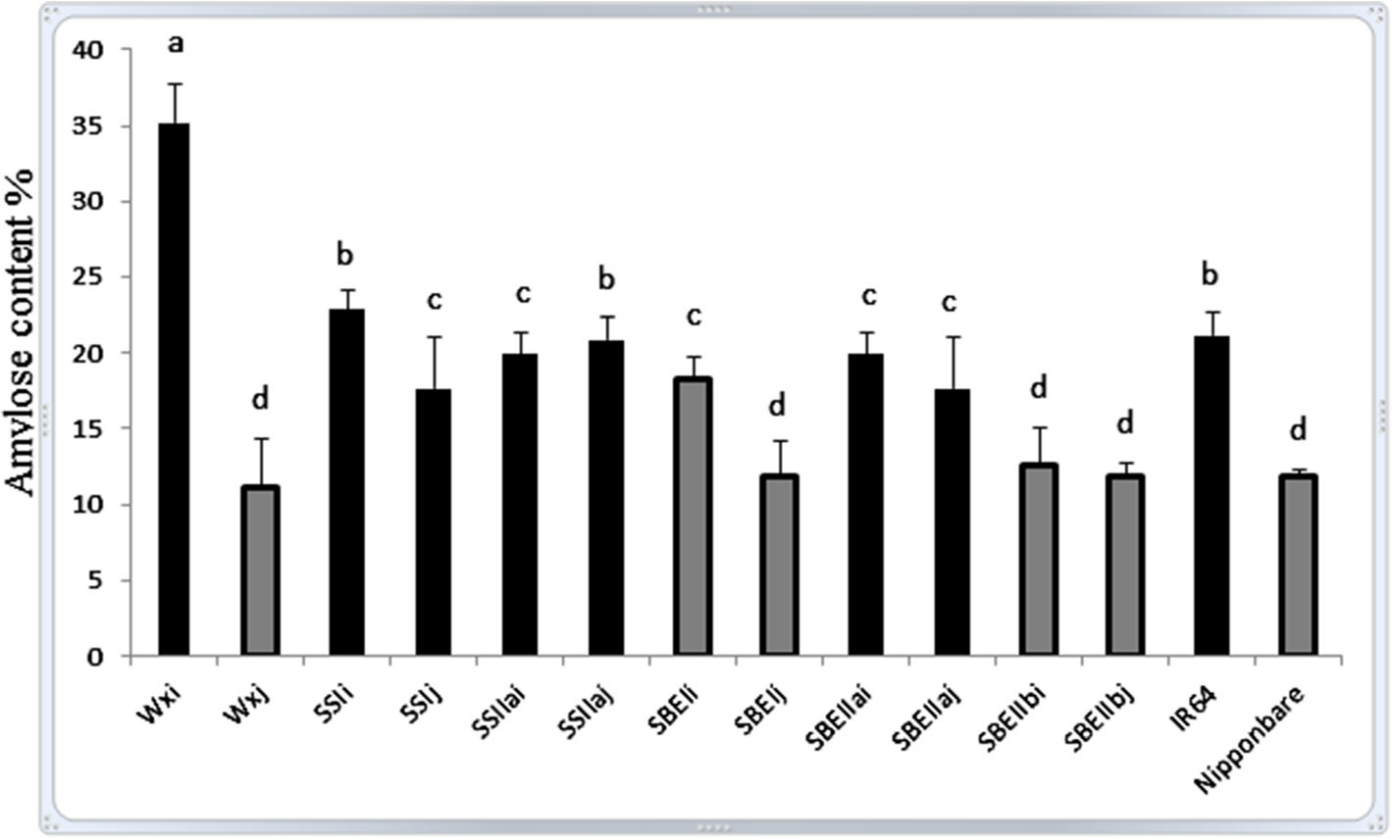
**Comparison of amylose content among 12 alleles of six starch synthetic genes using the SEC method.** Starches from five plants of each allele were isolated and analysed seperately. Two replicates were set up for each sample. The columns indicate starch AC of RIL grains. Black columns indicate those allelles containing *Wxi* allele and grey columns indicate those allelles containing *Wxj* allele. The error bars show the standard error of the mean. The identity of each column is indicated underneath. Columns with different letters are significantly different at *p* < 0.05.

Although the *Wxi* allele played a major role in the determination of increased AC, other alleles also showed impacts on AC. The range of AC of selected RILs was 11 ~ 35% approximately of rice grain starch (Figure 2). Six allele groups can be classified into two AC groups depending on the *Wx* allele. The *Wxi* allele group contained 17.7 ~ 35.3% amylose in starch (including *Wxi* allele and *SSI*, *SSIIa* and *SBEIIa* groups), whereas *Wxj* allele group contained 11.1 ~ 18.4% (including *Wxj* allele and *SBEI* and *SBEIIb* groups), with parental lines IR64 (21.2%) and Nipponbare (11.8%) fitting in their respective *Wx* groups. Between the *Wxi* allele group (containing average 22.0% amylose) and the *Wxj* allele group (containing average 13.1% amylose), the former contained ~9% significantly higher AC than the latter. Within *Wxi* allele group, starch samples from *Wxi* allele lines contained significantly higher AC (35.3%) than all other allele groups, and starch from *SSIi* (22.8%) and *SSIIaj* (20.9%) alleles contained higher AC than other four alleles (*SSIj*, *SSIIai*, *SBEIIai* and *SBEIIaj*) (Figure 2). Within *Wxj* allele group, *SBEIi* allele lines (18.4%) contained significantly higher AC than those from other four allele groups, among which AC was not significantly different.

### Chain length distribution (CLD) of debranched starch

**Figure 3:**
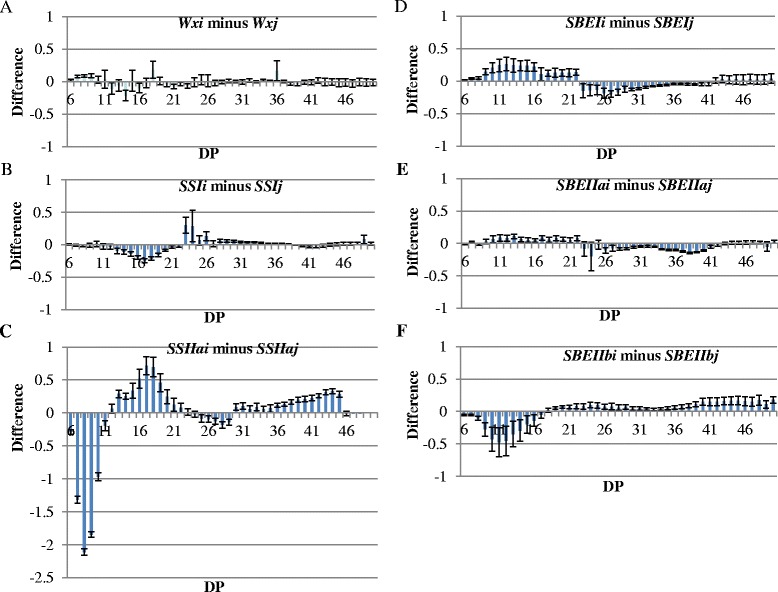
**Comparison of chain length distribution of debranched starch of six allele groups for six starch synthetic genes.** Starches from five plants of each allele were used for the analysis. Two replicates were set up for each sample. For each allele group, values of chain lengths for *japonica* allele lines were subtracted from values of chain length for the *indica* allele lines. **A**: *Wx*, **B**: *SSI*, **C**: *SSIIa*, **D**: *SBEI*, **E**: *SBEIIa*, **F**: *SBEIIb*. Each bar corresponds to the difference of a chain length in mole percentage. The error bars are the standard errors.

In this study, linear glucan chains of DP ≤ 24 comprised up to 80% mole of molecules in amylopectin of rice grains (Table 2). *SSIIai* played a major role, while *SBEIIbi* played a minor role in accumulating intermediate chains (DP12 ~ 22). The differences in % normalised distribution were obtained by subtracting the CLD of *japonica* allele from *indica* allele in each gene group, respectively (Figure 3). The profound difference in CLD was obtained in *SSIIa* allele group (Figure 3C). *SSIIai* allele starch contained fewer short chains at DP6 ~ DP11, more intermediate chains at DP12 ~ 22 and long chains at DP30 ~ 45 compared with that of *SSIIaj* allele starch. Limited variations were found in the other five gene groups, however, *SBEIIbi* allele showed up to 0.5% normalised distribution of fewer chains at DP10 ~ 13 than *SBEIIbj* allele (Figure 3F).

**Table 2:** **Chain length fractions of RILs of six starch biosynthetic enzyme allele groups**

**Genotype**	**∑DP ≤ 10** **%**	**∑DP ≤ 24** **%**	**∑DP > 24** **%**	**R**_**CL10/24**_ **×10** ^**−2**^
*Wxi*	10.3 ± 0.1^e^	78.4 ± 0.1^b^	21.7 ± 0.3^a^	13.1 ± 0.1^e^
*Wxj*	10.0 ± 0.1^e^	78.4 ± 0.1^b^	21.6 ± 0.4^a^	12.7 ± 0.1^e^
*SSIi*	10.8 ± 0.1^d^	78.8 ± 0.1^b^	21.2 ± 0.2^b^	13.6 ± 0.1^d^
*SSIj*	10.8 ± 0.1^d^	79.6 ± 0.1^a^	20.5 ± 0.2^b^	13.5 ± 0.1^d^
*SSIIai*	10.6 ± 0.1^d^	78.3 ± 0.1^b^	21.7 ± 0.2^b^	13.5 ± 0.1^d^
*SSIIaj*	15.9 ± 0.1^b^	76.7 ± 0.1^c^	23.3 ± 0.4^a^	20.7 ± 0.1^b^
*SBEIi*	9.8 ± 0.1^e^	79.3 ± 0.1^a^	20.7 ± 0.5^b^	12.4 ± 0.1^f^
*SBEIj*	9.5 ± 0.1^f^	77.7 ± 0.1^b^	22.4 ± 0.3^a^	12.2 ± 0.1^f^
*SBEIIai*	10.8 ± 0.1^d^	79.6 ± 0.1^a^	20.5 ± 0.2^b^	13.5 ± 0.1^d^
*SBEIIaj*	10.6 ± 0.1^d^	78.3 ± 0.1^b^	21.7 ± 0.2^b^	13.5 ± 0.1^d^
*SBEIIbi*	10.2 ± 0.2^e^	78.5 ± 0.2^b^	21.5 ± 0.8^a^	12.9 ± 0.2^e^
*SBEIIbj*	11.1 ± 0.3^d^	81.2 ± 0.3^a^	18.8 ± 1.2^c^	13.7 ± 0.2^d^
Parent control				
IR64	12.8 ± 0.1^c^	79.7 ± 0.2^a^	19.3 ± 0.3^b^	16.1 ± 0.1^c^
Nipponbare	22.9 ± 0.0^a^	80.1 ± 0.1^a^	19.9 ± 0.2^b^	28.6 ± 0.1^a^

Values are mean values of percentages of the mole basis of five different RIL lines of each allele with two replicates. R_CL10/24_ is the ratio of ∑DP ≤ 10/∑DP ≤ 24. IR64 and Nipponbare are the parental lines. Values with different characters are significantly different at *p* < 0.01.

Based on Nakamura’s study (Nakamura et al. 2002), *indica* and *japonica* type amylopectin from the majority of cultivated Asian rice strains can be distinguished by the ratio of ∑DP ≤ 10 to ∑DP ≤ 24 fractions (R_CL10/24_) of CLD. Our present study showed the R_CL10/24_ value was 0.16 for IR64 and 0.29 for Nipponbare (Table 2), and most of the selected RILs were smaller than 0.14, suggesting that they were *indica* type. With the only exception of *SSIIaj* allele, its R_CL10/24_ value was slightly over 0.20. The highest value of *SSIIaj* resulted from a high percentage of short chain fraction (∑DP ≤ 10, significantly higher than that of *SSIIai* allele, and 43 ~ 67% higher than the other RILs) and low percentage of short and intermediate chain fraction (∑DP ≤ 24, statistically significantly lower than that of *SSIIai* allele) in the starch. In terms of the longer chain fraction (∑DP > 24), *SSIIaj* was the highest but not significantly different to a few other alleles in the group (Table 2). Besides *SSIIaj*, *SBEIIbj* allele was also detected with more short chains in starch than the counterpart-allele *SBEIIbi*.

### Starch paste viscosity

By analysing RVA characteristics pasting properties of starch were determined as reported in previous studies (Sasaki et al. 2000; Han and Hamaker 2001; Chen et al. 2003). The RVA characteristics involved in this study were peak viscosity (PV), trough (Tr), breakdown (BD), final viscosity (FV), setback (SB) and peak time (PT). The values of these characteristics were statistically analysed and shown in Table 3. The RVA result revealed that *i* and *j* alleles from each of six allele groups contributed to parameters of starch paste viscosity properties differentially.

**Table 3:** **Viscosity properties of RIL wholemeal flours analysed using RVA**

**Genotype**	**Peak viscosity ** **(RVU)**	**Trough ** **(RVU)**	**Breakdown ** **(RVU)**	**Final viscosity ** **(RVU)**	**Setback ** **(RVU)**	**Peak time ** **(min)**
*Wxi*	227.7 ± 6.0^b^	109.5 ± 8.1^a^	118.1 ± 10.1^b^	243.6 ± 7.1^a^	134.0 ± 1.9^b^	8.7 ± 0.08^a^
*Wxj*	283.6 ± 2.5^a^	121.1 ± 1.2^a^	162.4 ± 3.1^a^	213.0 ± 2.4^b^	91.9 ± 1.9^d^	8.5 ± 0.02^b^
*SSIi*	210.1 ± 3.7^c^	79.1 ± 3.3^b^	131.0 ± 6.2^b^	194.2 ± 6.8^b^	115.2 ± 3.6^c^	8.3 ± 0.12^b^
*SSIj*	215.6 ± 6.2^c^	112.1 ± 5.1^a^	103.6 ± 1.7^c^	235.0 ± 7.9^a^	122.9 ± 3.5^b^	8.9 ± 0.02^a^
*SSIIai*	246.8 ± 4.2^b^	109.0 ± 3.4^a^	137.8 ± 5.0^b^	232.5 ± 5.8^a^	123.5 ± 2.7^b^	8.8 ± 0.10^a^
*SSIIaj*	181.3 ± 6.0^d^	112.9 ± 7.6^a^	68.4 ± 2.9^d^	264.4 ± 12.9^a^	151.5 ± 5.4^a^	8.8 ± 0.06^a^
*SBEIi*	252.6 ± 8.1^b^	107.9 ± 2.7^a^	144.7 ± 5.4^b^	204.4 ± 3.0^b^	96.5 ± 0.4^d^	8.5 ± 0.00^b^
*SBEIj*	240.1 ± 5.0^b^	118.7 ± 2.6^a^	121.4 ± 2.4^b^	225.1 ± 3.6^b^	106.4 ± 1.5^c^	8.5 ± 0.02^b^
*SBEIIai*	215.6 ± 6.2^c^	112.1 ± 5.1^a^	103.6 ± 1.7^c^	235.0 ± 7.9^a^	122.9 ± 3.5^b^	8.9 ± 0.02^a^
*SBEIIaj*	246.8 ± 4.2^b^	109.0 ± 3.4^a^	137.8 ± 5.0^b^	232.5 ± 5.8^a^	123.5 ± 2.7^b^	8.8 ± 0.10^a^
*SBEIIbi*	291.8 ± 2.6^a^	106.3 ± 3.4^a^	185.6 ± 5.6^a^	200.5 ± 3.2^b^	94.3 ± 2.7^d^	8.4 ± 0.04^b^
*SBEIIbj*	256.3 ± 3.9^b^	96.7 ± 4.5^b^	159.6 ± 7.5^a^	184.3 ± 3.6^c^	87.6 ± 2.0^d^	8.4 ± 0.12^b^
Parent control						
IR64	267.4 ± 1.2^a^	97.9 ± 2.3^a^	169.5 ± 3.10^a^	216.2 ± 2.8^b^	118.3 ± 1.7^c^	8.2 ± 0.08^c^
Nipponbare	214.6 ± 10.0^c^	97.0 ± 4.0^b^	117.6 ± 6.05^b^	195.1 ± 5.7^b^	98.1 ± 1.7^d^	8.5 ± 0.02^b^
l.s.d.	29.11	23.47	28.47	32.24	14.20	0.35

RVU is rapid viscosity unit. Each value is the mean of 3 biological replicates. Mean values within columns with difference letters are significantly different at *p* < 0.01.

IR64 and Nipponbare showed significant differences in all parameters except for FV (Table 3). RILs in *Wx*, *SSIIa*, *SBEIIa* and *SBEIIb* allele groups had significantly distinct PV between *i* and *j* alleles, respectively. PV values of *Wxj* and *SSIIai* alleles were ~60 RVU higher than *Wxi* and *SSIIaj* alleles, whereas the increase in *SBEIIaj* and *SBEIIbi* alleles was smaller (31 ~ 35 RVU) comparing to *SBEIIai* and *SBEIIbj* alleles. Tr was the least diverse RVA parameter in this analysis, showing only about 30 and 10 RVU significant increase in *SSIj* and *SBEIIbi* alleles comparing to their counterpart-alleles, respectively. Significant differences in BD were obtained in *Wx*, *SSI*, *SSIIa* and *SBEIIa* allele groups, with the greatest (~70 RVU) between *SSIIai* and *SSIIaj* alleles. Compared to their counterparts, the values of BD were increased by ~40 RVU in *Wxj* allele, and ~30 RVU in *SSIi* and *SBEIIaj* alleles. In terms of FV, significant variations were found in *Wx*, *SSI* and *SBEIIb* allele groups, with the highest (~40 RVU) between *SSIi* and *SSIj* alleles, ~30 and 15 RVU in *Wx* and *SBEIIb* allele groups, respectively. The significant variations of SB were 42 RVU in *Wx* group and 28 RVU in *SSIIa* group. Less than 10 RVU increase of SB in both *SSIj* and *SBEIj* alleles were also significantly different. Significant differences in PT were determined between *SSI* alleles (0.6 min) and *Wx* group (0.2 min). The PT values were mainly clustered into the two *Wx* alleles groups, while IR64 was the lowest. Notably, the *SSIIaj* allele starch exhibited remarked differences in paste viscosities from all the others including parental controls.

### Starch thermal properties

Consistent with previous studies, two primary peaks were observed in DSC curves for rice wholemeal samples: the first one was the initial gelatinization peak at 65 ~ 85°C of amylopectin; the second one at around 105°C was the amylose-lipid complex dissociation peak (Biliaderis et al. 1985). Onset, peak and end temperatures of the first peak were named as ^1^To, ^1^Tp and ^1^Te, and those of the second peak as ^2^To, ^2^Tp, ^2^Te. ^1^ΔH was named for the amylopectin gelatinization enthalpy, and ^2^ΔH for the dissociation enthalpy of amylose-lipid complexes. As shown in Table 4, the DSC result indicated that *SSIIaj* allele had a major effect, while *Wxi* allele played a minor role in decreasing ^1^Tp. However, *SSIj*, *SSIIaj* and *Wxi* alleles increased ^2^ΔH.

**Table 4:** **Thermal characteristics of RIL flours determined using DSC**

**Genotype**	^**1**^ **To (°C)**	^**1**^ **Tp (°C)**	^**1**^ **Te (°C)**	^**1**^ **ΔH (J/g)**	^**2**^ **To (°C)**	^**2**^ **Tp (°C)**	^**2**^ **Te (°C)**	^**2**^ **ΔH (J/g)**
*Wxi*	73.4 ± 0.6^b^	80.1 ± 0.3^c^	87.1 ± 0.6^c^	3.1 ± 0.3^a^	97.7 ± 0.4^a^	104.1 ± 0.6^a^	109.1 ± 1.0^a^	0.42 ± 0.08^c^
*Wxj*	76.4 ± 0.4^a^	83.0 ± 0.4^b^	89.6 ± 0.0^a^	3.3 ± 0.0^a^	99.8 ± 0.5^a^	103.5 ± 0.5^a^	104.3 ± 1.8^a^	0.21 ± 0.02^d^
*SSIi*	71.8 ± 2.5^b^	77.6 ± 0.4^e^	85.9 ± 0.7^d^	2.6 ± 0.2^b^	98.9 ± 1.1^a^	102.9 ± 0.7^a^	106.5 ± 1.5^a^	0.36 ± 0.05^c^
*SSIj*	73.1 ± 0.3^b^	79.3 ± 0.3^c^	86.1 ± 0.5^c^	2.9 ± 0.2^b^	99.4 ± 1.1^a^	103.7 ± 1.3^a^	105.7 ± 2.5^a^	0.60 ± 0.14^b^
*SSIIai*	71.6 ± 0.5^b^	78.7 ± 0.1^d^	86.2 ± 0.3^c^	2.7 ± 0.1^b^	97.3 ± 0.8^b^	106.4 ± 1.3^a^	109.6 ± 1.7^a^	0.70 ± 0.06^b^
*SSIIaj*	60.5 ± 0.3^d^	67.9 ± 0.5^g^	77.2 ± 1.1^f^	2.8 ± 0.1^b^	99.7 ± 3.8^a^	104.2 ± 4.7^a^	109.6 ± 3.6^a^	1.20 ± 0.05^a^
*SBEIi*	76.5 ± 0.4^a^	83.4 ± 0.4^a^	89.9 ± 0.4^a^	2.9 ± 0.0^b^	99.0 ± 1.9^a^	104.5 ± 1.2^a^	109.5 ± 0.8^a^	0.42 ± 0.01^c^
*SBEIj*	77.4 ± 0.8^a^	84.4 ± 0.3^a^	90.9 ± 0.1^a^	3.2 ± 0.1^a^	101.0 ± 0.3^a^	105.7 ± 0.4^a^	108.0 ± 1.4^a^	0.50 ± 0.04^c^
*SBEIIai*	73.1 ± 0.3^b^	79.3 ± 0.3^c^	86.1 ± 0.5^c^	2.9 ± 0.2^b^	99.4 ± 1.1^a^	103.7 ± 1.3^a^	105.7 ± 2.5^a^	0.60 ± 0.14^b^
*SBEIIaj*	71.6 ± 0.5^b^	78.7 ± 0.1^d^	86.2 ± 0.3^c^	2.7 ± 0.1^b^	97.3 ± 0.8^b^	106.4 ± 1.3^a^	109.6 ± 1.7^a^	0.70 ± 0.06^b^
*SBEIIbi*	76.9 ± 1.0^a^	83.4 ± 0.6^b^	90.4 ± 0.6^a^	3.5 ± 0.0^a^	101.6 ± 1.4^a^	105.7 ± 0.5^a^	108.5 ± 2.1^a^	0.28 ± 0.01^d^
*SBEIIbj*	77.3 ± 0.2^a^	83.3 ± 0.3^b^	89.2 ± 0.4^b^	3.2 ± 0.0^a^	98.0 ± 0.1^a^	104.1 ± 0.5^a^	107.7 ± 2.0^a^	0.22 ± 0.03^d^
Parent control								
IR64	72.9 ± 0.1^b^	80.2 **±** 0.1^c^	87.5 ± 0.2^c^	2.7 ± 0.1^b^	97.65 ± 0.94^a^	102.5 ± 0.9^a^	106.9 ± 0.4^a^	0.38 ± 0.04^c^
Nipponbare	65.2 ± 1.4^c^	74.9 ± 0.1^f^	83.0 ± 0.1^e^	2.7 ± 0.1^b^	96.97 ± 0.93^b^	101.7 ± 0.4^b^	101.8 ± 1.8^b^	0.34 ± 0.01^c^
l.s.d.	2.77	1.04	1.48	0.42	4.23	4.61	5.46	0.17

^1^To, ^1^Tp and ^1^Te are onset, peak and end gelatinization temperatures, and ^1^ΔH is the gelatinization enthalpy at the amylopectin gelatinization peak. ^2^To, ^2^Tp, and ^2^Te are onset, peak and end dissociation temperatures of amylose-lipid complexes, and ^2^ΔH is the dissociation enthalpy at the dissociation peak of amylose-lipid complexes. Each value is the mean of three biological replicates. Mean values within the same column with different letters are significantly different at *p* < 0.01.

Regarding amylopectin gelatinization temperatures, IR64 had significantly higher values in ^1^To, ^1^Tp and ^1^Te than Nipponbare, whereas the RILs exhibited divergent values. *Wxj* allele group observed ~3°C higher ^1^To than those from *Wxi* allele group. There was barely any variation in ^1^To among different alleles within *Wxi* allele group except *SSIIaj* allele having ~11°C decrease compared with *SSIIai* allele. In terms of ^1^Tp, *Wxj* allele group overall was ~3°C higher than *Wxi* allele group. Within *Wxi* allele group, *SSIIai* was ~11°C higher than *SSIIaj*, while *SSIj* and *SBEIIai* were ~1 and 0.5°C higher than *SSIi* and *SBEIIaj* alleles, respectively. For ^1^Te, *Wxj* allele group overall was ~2°C higher than *Wxi* allele group. There was ~9°C increase in *SSIIai* allele comparing to *SSIIaj* allele. In terms of ^1^ΔH, *Wxj* allele group was ~0.4 J/g higher than *Wxi* allele group, except for *SBEIi* allele 0.3 J/g lower than the counterpart-allele.

Regarding dissociation temperatures of amylose-lipid complexes, IR64 wholemeal had significantly higher values of ^2^To, ^2^Tp and ^2^Te than Nipponbare. In terms of ^2^To, the only significant variations were observed in *SSIIaj* and *SBEIIai* alleles which both had ~2°C increase compared with their counterpart-alleles. In terms of ^2^ΔH, *SSIIaj* and *SSIj* alleles were up to 0.5 J/g and 0.24 J/g higher than *SSIIai* and *SSIi* alleles, respectively. *Wxi* allele group overall exhibited higher energy than *Wxj* allele group. Within *Wxi* group, *SSIIaj* and *Wxi* were the highestand lowest, whereas in *Wxj* allele group *SBEIi* allele had higher ^2^ΔH than the others. Interestingly, similar to the observation in RVA examination, *SSIIaj* allele starch also showed remarked differences in thermal properties from others.

### Protein analyses in starch granules of mature grains

**Figure 4:**
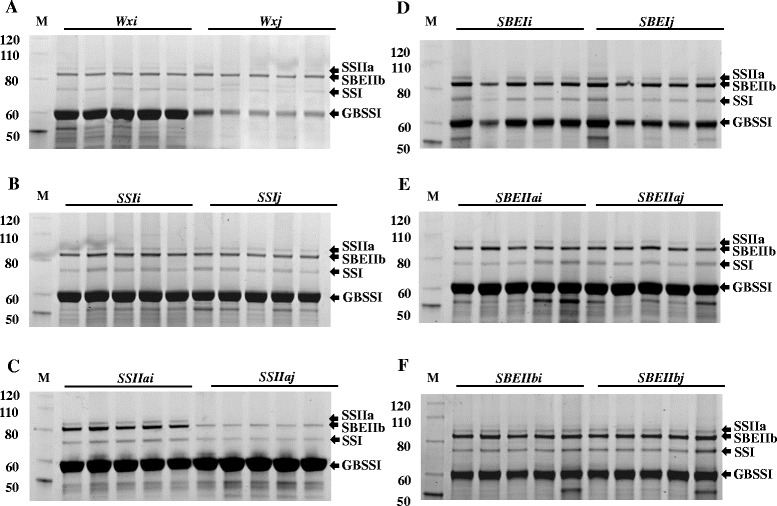
**Analysis of starch GBPs in mature rice grain starch of RILs from six allele graoups of six starch synthetic genes by SDS-PAGE.** Starches from five RIL lines of each allele were used. Section **A**: *Wx* allele group, **B**: *SSI* allele group, **C**: *SSIIa* allele group, **D**: *SBEI* allele group, **E**: *SBEIIa* allele group, **F**: *SBEIIb* allele group. The molecular sizes are labelled on the left of protein marker bands in kDa. The identity of each protein band in the samples is indicated on the right side of the pictures by an arrow head.

**Figure 5:**
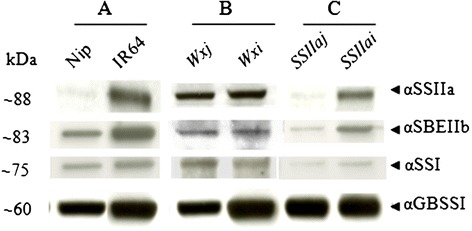
**Immunodetection analysis of GBPs of purified starch from mature rice grains. A**, parental lines; **B** and **C**, selected RIL lines. The names of alleles are labelled on top of each lane for *Wxj* (3-14-13), *Wxi* (3-14-12), *SSIIaj* (3-6-1) and *SSIIai* (3-6-9). The estimated molecular weight of protein bands are shown on the left. The protein bands detected by various antibodies are indicated by arrows on the right.’Nip’ is the abbreviation for Nipponbare.

The analysis of GBPs prepared from purified starch of mature rice grains showed that four major protein bands with 60 kDa and above were detected in most of the RILs (Figure 4). In parental lines, the top bands at ~88 kDa were identified as SSIIa, ~83 kDa as SBEIIb, ~75 kDa as SSI and ~60 kDa as GBSSI by immunoblotting using specific antibodies (Figure 5).

Comparing the two parental lines, IR64 contained a higher amount of GBSSI, SSI, SSIIa and SBEIIb in the GBPs than Nipponbare, and SSIIa was barely detectable by immunodetection in Nipponbare (Figure 5A). Consistent with parental lines, GBSSI abundance of *Wxi* allele was significantly higher than that of *Wxj* allele, while the abundance of SSI, SSIIa and SBEIIb in *Wxi* and *Wxj* alleles remained at same levels, respectively (Figures 4A, 5B). In *SSIIa* alleles, the amount of SSI, SSIIa and SBEIIb protein was significantly decreased in *SSIIaj* allele (Figure 5C). Similarly, only faint bands of SSIIa were detected in *SSIIaj* RILs (Figure 4C), whereas GBSSI remained at the same level. No significant changes were observed in abundance of the four proteins in SDS-PAGE gels of other groups.

## Discussion

In this work, six starch synthetic gene alleles were used for genotyping progenies from one crossing line between *indica* and *japonica* rice. We aimed at understanding the roles of those alleles on starch properties of rice grain. The results showed that both amylose content and amylopectin structure were affected by multiple gene alleles, although some of them play major roles, others play minor roles. However, as matter of time, the roles of other genes involved in starch biosynthesis could not be studied, eg. SSIIIa gene affects amylose content (Gao et al. 1998; Fujita et al. 2007; Li et al. 2011) and starch debranching enzymes (Kubo et al. 1999). Therefore, more research work is required to define the functions of other genes on starch property variation between *indica* and *japonica* rice.

### The relation between amylose content and starch functional properties

Starch AC has been reported to be negatively correlated with the gelatinization (^1^Tp) and pasting properties (PV) of starches (Sasaki et al. 2000; Chen et al. 2003; Varavinit et al. 2003; Park et al. 2007). In the current study, among the six allele groups, the *Wxi* allele is determined as a major allele for AC increase. This result for *Wxi* allele is consistent with early works on *Wx*^*a*^, a high mRNA expressing *Wx* allele, which has been identified as a major gene controlling amylose content (Sano 1984; Wang et al. 1995). *Wx*^*a*^ allele explains most of the difference in increasing amylose content, decreasing the PV and BD of RVA in an *indica* rice variety, which greatly affects the quality of cooked rice (Umemoto et al. 2008). In addition, the current study also shows the impact of *SSIi*, *SSIIaj* and *SBEIi* alleles in determining difference of amylose content in RIL lines comparing to their corresponding allele pairs. The nature of high AC for *Wxi* allele group remains to be studied further. Analysis of a complete set of 64 potential combinations may explain the nature of its high AC. In the perspective of starch functional properties, high values were determined for paste and thermal properties in *Wxj* allele and other three relatively low amylose alleles (*SSIj*, *SSIIai* and *SBEIj*) compared to their counterpart-alleles. This correlation again confirms starch paste and thermal properties are negatively regulated by the ratio of amylose produced in the grains.

### The relation between amylopectin chain length distribution and starch functional properties

Previous studies showed that short chains (DP 6 ~ 10) and intermediate (DP 11 ~ 24) chains of amylopectin are the major components of amylopectin structure in rice endosperms (Hizukuri 1985, 1986; Hizukuri et al. 1989). The variations in short and intermediate chains were related with starch pasting and thermal property changes (Park et al. 2007). Thus, it is interesting to investigate the impact of *indica* and *japonica* alleles on the relation of amylopectin CLD and starch functional properties. Since R_CL10/24_ was the same between *Wxi* and *Wxj* alleles, it suggests Wx protein was not involved in the synthesis of short chains in amylopectin. The roles of *SSIj* allele in increasing starch R_CL12/24_ value and starch properties in rice grain were reported by early researchers (Takemoto-Kuno et al. 2006) through comparing starches from progeny lines derived from the crossing population between *indica* and *japaonica* rice. However, such increasing starch R_CL10/24_ value was not observed from *SSIj* allele in this work although starch from *SSIj* allele contained more short chains less than DP 24 comparing to that from *SSIi* allele. The discrepancy between early publication (Takemoto-Kuno et al. 2006) and our current work may be due to the different varieties used to develop RIL population or the effects from *SSIj* and *SBEIIbj* combination. Umemoto et al. (2008) studied a near-isogenic line-NIL (*SSI*^*k*^) containing a *SSI* allele from an indica rice on a japonica rice background and concluded the variation in *SSI* alleles between indica and japonica rice hardly affected starch peak viscosity and gelatinization temperatures. The *SSIIaj* allele starch showing more short chains and less intermediate chains was determined with a remarkably high R_CL10/24_. This variation led to distinctive characteristics of starch properties with the lowest PV, BD, and lowest gelatinization temperatures. Despite other alleles within *Wxi* allele group showed lower PV and BD than IR64, they were comparable in R_CL10/24_ values. Consequently, these alleles barely contribute to the variations of RVA and DSC characteristics within the *Wxi* allele group. Within the *Wxj* allele group, the *SSIIai* allele was prevalent showing similar CLD patterns, which was significantly different from Nipponbare. The *SBEIIbj* allele had a significantly higher R_CL10/24_ value comparing to *SBEIIbi*, but it was much lower than that of Nipponbare. The *SBEIIbj* allele had abundant short chains in amylopectin with lower PV, Tr and lower FV and lower ^1^Te than *SBEIIbi* allele.

Given all the analyses above, R_CL10/24_ is negatively correlated with starch viscosity and thermal gelatinization properties. An increase of R_CL10/24_ can reduce the PV values of RVA and gelatinization temperature of DSC due to the increase of short chains proportion and/or decrease of intermediate chains proportion. *SSIIaj* is a major allele and *SBEIIbj* is a minor allele in the determination of R_CL10/24_ in this study. Interestingly, starch PV and gelatinization temperatures of the *SSIIaj* allele are even lower than Nipponbare, though the *SSIIaj* allele R_CL10/24_ is smaller. This is because *SSIIaj* allele combines with *Wxi* allele in the corresponding RILs. Such changes of starch properties were also reported by Umemoto et al. (2008) through study of near-isogenic line-NIL (Wxa) which contained a chromosome segment containing a Wx allele from an indica rice (Kasalath) on a janonica (Nipponbare) rice background. Our analyses here demonstrate that both high amylose content and high R_CL10/24_ regulate starch properties by lowering starch PV and gelatinization temperatures. *SSIIaj* and *SBEIIbj* alleles contribute to high R_CL10/24._ Therefore, the novel starch functional properties of *SSIIaj* allele is probably the result of the combination of major alleles, *SSIIaj* (high R_CL10/24_) and *Wxi* (high AC), with minor alleles, *SBEIi* and *SBEIIbj*.

### The relation between starch structure and starch granule bound proteins

The *Wx* allele group showed significant variation in the abundance of GBSSI in the starch granule of rice grains. RILs with the *Wxj* allele contained a relatively low abundance of GBSSI and a low level of starch AC compared with those with the *Wxi* allele. This was consistent with previous studies which demonstrated that *Wxj* allele produces low levels of mature enzymes (Cai et al. 1998; Hirano et al. 1998), and rice varieties with *Wxi* and *Wxj* alleles have different levels of starch AC in rice grains (Sano 1984; Wang et al. 1995). However, the change of GBSSI abundance inside starch granules did not affect the abundance of other starch biosynthetic enzymes in starch granules. SSIIa was the other protein with varied abundance in starch granules between *indica* and *japonica* alleles. Moreover, *SSIIaj* allele starch contained a reduction of SSI and SBEIIb. According to Umemoto et al. (2004), the SSIIa protein from IR64 and Nipponbare differ by two amino acid substitutions, the protein from Nipponbare has reduced SSIIa activity. Furthermore, the reduced SSIIa activity resulted in the decrease of SSIIa abundance in starch granules, and ultimately produced amylopectin with increased R_CL10/24_ values and slightly increased AC. Although *SSIi, SBEIIaj* and *SBEIIbj* alleles showed limited effects on starch structures and properties, the changes in the expression levels of these proteins were hardly detected from current study.

A recent study in rice and maize suggested that the majority of GBPs are involved in starch biosynthesis (Koziol et al. 2011). It indicated that the GBPs are important for the starch structure and properties. In our study, the remarkable changes in GBPs of *SSIIa* and *Wx* alleles observed in starch granules is associated with the pronounced changes in starch structure and functional properties as described earlier. This is consistent with the observations by numerous previous studies on single or double starch biosynthetic gene-recessive mutants of SSI, SSIIa and SBEIIb proteins, not only in rice but other cereals (Morell et al. 2003; Nakamura et al. 2005a; Kosar-Hashemi et al. 2007; Grimaud et al. 2008; Li et al. 2011). Therefore, detecting significant changes in the abundance and composition of GBPs is an alternative approach to screen variants with favorable starch structure and functional properties.

## Conclusions

This study showed that rice *Wxi* and *SSIIaj* alleles are major contributors, whereas *SSIi*, *SBEIi*, *SBEIIai* and *SBEIIbj* alleles are minor contributors to rice starch properties between *indica* and *japonica* rice. The combination of *SSIIaj* and *Wxi* with *SBEIIbj* and *SBEIi* alleles has accumulative effects lowering PV and gelatinization temperature, which leads to the novel functional properties of *SSIIaj* allele starch in the study. Starch AC and the amylopectin CLD determine a number of starch functional properties. The R_CL10/24_ in amylopectin is negatively correlated with PV and BD of paste property and GTs of thermal property. Moreover, we suggest that the alteration of GBPs inside starch granules is a potential indicator for significant changes in starch structure and functional properties. A future study involving 64 possible combinations for the 6 gene groups in a common genetic background in rice will lead to an in-depth understanding of starch structures and functional properties.

## Methods

### Plant materials

Two rice (*Oryza sativa*) parental lines [cultivars IR64 (*indica* pollen donor) and Nipponbare (*japonica* acceptor)] were used for producing a RIL population. Four F2 seeds from one F1 seed were produced (kindly provided by Dr. Narayana Upadhyaya). Only one F2 plant was used for producing all RILs in this study. The detailed steps for selecting RILs for six allele groups are listed in Figure 1. All plants including RILs and their parental lines were grown in a glass house of a plant growth facility at CSIRO Agriculture Flagship (Canberra, ACT, Australia) at 27°C under natural light. Mature seeds were harvested for starch preparation, and starch structure and properties analysed.

### Genomic DNA preparation

The genome DNA was extracted with a quick DNA extraction method. Fresh young leaves of 2 cm length were sampled into microfuge tubes on ice and immediately ground in 66 μl of 0.5% NaOH using a micropestle. Samples were then incubated in a boiling water bath for one minute, and diluted with 120 μl of 0.1 M Tris–HCl, pH8.0. DNA was precipitated by adding 180 μl isopropanol and 18 μl 3 M sodium acetate, pH5.2 to sample tubes and gently mixed by reversing the tube, and incubated at room temperature for 5 min. The supernatant was discarded after the centrifugation for 30 min at 3000 g, and the pellet was air dried at room temperature for 1 min and washed with 180 μl of 70% (V/V) ethanol. After centrifugation at 3000 g, the genomic DNA in the pellet was then dissolved in 50 μl MilliQ water.

### Genotyping of selected genes

The genotypes of RILs for the selected genes in the study were identified based on a PCR approach using published markers (Yan et al. 2007; Tian et al. 2010). For each allele, five individual plants were selected for generating data for statistical analysis. Due to the limitation of material availability, only three of five plants with more grains of each allele were used for starch property characterization. Alleles of *indica* derived from IR64 were assigned as *Wxi*, *SSIi*, *SSIIai*, *SBEIi*, *SBEIIai* and *SBEIIbi*, and corresponding *japonica* alleles derived from Nipponbare were named as *Wxj*, *SSIj*, *SSIIaj*, *SBEIj*, *SBEIIaj* and *SBEIIbj*. The alleles were segregated in different combinations during subculture, and F5 homozygotes were used for further analyses.

Three F3 RILs (3–5, 3–6 and 3–14) from one F2 line were used for the further selection of different combinations of the 6 genes by self-pollination due to their homozygosity and heterozygosity of six different genes in three F3 lines. Line 3–5 was homozygous for *SSIi*, *SSIIai* and *SBEIIai* and heterozygous at *Wx*, *SBEI* and *SBEIIb* loci. Among the F4 selfing progeny of F3 line 3–5, lines 3-5-1, 3-5-2 and 3-5-15 were homozygous for *SBEIIbi* and *Wxj*, *SBEIi* and *Wxj*, *SBEIi* and *Wxi*, respectively. They were further grown to obtain homozygous lines for *indica* and *japonica* type for *SBEI* and *SBEIIb* genes*.* In the F5 progeny, line 3-5-1-2 and line 3-5-1-8 were homozygous at *SBEI*, with *SBEIi* and *SBEIj*, respectively; line 3-5-2-1 and line 3-5-15-11 were homozygous at *SBEIIb*, with *SBEIIbj*; line 3-5-2-2 was homozygous for *SBEIIbi*. Line 3–6 was homozygous for *Wxi*, *SSIj* and *SBEIi* and heterozygous at *SSIIa*, *SBEIIa* and *SBEIIb* loci. Among the F4 progenies of line 3–6, three lines were homozygous for *SSIIaj, SBEIIaj* and *SBEIIbj* (line 3-6-1), *SSIIai*, *SBEIIaj* and *SBEIIbj* (line 3-6-9), and *SSIIai*, *SBEIIai* and *SBEIIbj* (line 3-6-20), respectively. RIL line 3–14 was homozygous at *SSIi*, *SSIIai*, *SBEIi*, *SBEIIaj* and *SBEIIi*, and heterozygous at *Wx. Wxi* and *Wxj* alleles were obtained in lines 3-14-12 and line 3-14-13 lines in the F4 progeny.

### Starch preparation

Whole grains of each line (approximately 100 ~ 150 mg) were ground in a capsule with ball bearing using an ESPE CapMix™ (model 3 M, AU). Grinding was done for 30 sec three times. The wholemeal flour was then washed with 0.005% NaOH by vigorous vortexing for 2 minutes followed by filtration through 0.5 mm nylon sieves. Each sample was then washed three times by vortex mixing and centrifugation at 5000 g for 5 min. The starch pellet was resuspended in a phosphate buffer (50 mM, pH7.5) containing proteinase K (50 μg/ml) and incubated at 37°C for 2 hrs. After 5 min centrifugation at 5000 g, the pellet was suspended in water and centrifuged. Thus water wash was repeated three times. The starch pellet was then washed with acetone, centrifuged and the residual starch was air dried at 37°C overnight.

### Amylose content assay

The amylose content of samples was determined by analysing debranched starches using size-exclusion chromatography (SEC) with Ultrahydrogel (Waters, Milford, MA, USA) as described previously (Butardo et al. 2011). Pullulan standards (Shodex P-82) calibrated with the Mark–Houwink–Sakaruda equation were used for the estimation of the molecular weight from the elution time (Castro et al. 2005; Ward et al. 2006). Samples were prepared and analysed in triplicate.

### Chain length distribution of debranched starch

Samples were prepared as previously described with modifications (O’Shea and Morell 1996). For each sample, rice wholemeal (10 mg) was first presoaked in 40 μl ethanol and then incubated with 200 μl NaOH (0.25 M) and 600 μl MilliQ water in a boiling water bath with stirring for 10 min. The samples were then neutralized in the sodium acetate buffer (0.2 M sodium acetate and 3.6% V/V glacial acetic acid). Afterwards, 10 μl of isoamylase (1000 U/ml, Megazyme) was added to each sample to debranch the starch by incubating at 50°C for 2 h. The samples were boiled again for 10 min to denature isoamylase and centrifuged at 15000 g for 10 min. A 50 μl aliquot of supernatant was taken and dried down in a speed vacuum. The dried samples were dissolved in APTS (8-Aminopyrene-1,3,6-trisulfonic acid trisodium salt, BECKMAN COULTER) sodium cyanoborohydride buffer (containing 5 mg APTS labeling dye in 48 μl 15% acetic acid) and incubated overnight at 50°C. After the incubation, the samples were boiled in a urea solution (6 mM) for 1 min and filtered through Wizard miniColumns (Promega). Determination of the chain length distribution (CLD) of amylopectin was conducted by fluorescence-activated CE as previously described (O’Shea and Morell 1996).

### Starch paste viscosity

The polished rice grains (about 5 g) were ground into flour in a stainless steel capsule on a ball bearing machine (model MM300, MEP instruments Pty Ltd, NSW, AU) for starch property analysis. A Rapid Visco Analyzer (model RVA-4SA, Newport Scientific, Sydney, NSW, AU) was used to determine starch paste viscosity parameters of the selected RILs. Three grams of rice grain wholemeal for each of three plants of each RIL were used for analyses. The program setting for the RVA comprised the following stages: hold at 60°C for 2 min, heat to 95°C over 6 min, hold at 95°C for 4 min, cool to 50°C over 4 min, and hold at 50°C for 4 min. The software Thermocline (Newport Scientific PtyLtd, Warriewood, NSW, AU) was used for data collection and analysis.

### Differential scanning calorimetry

A Differential Scanning Calorimeter (model Pyris 1DSC, Perkin Elmer, Norwalk, CT, US) was used to determine the calorimetry profiles of flour samples of 3 different plants with triplicate for each RIL. For each line, 60 mg of flour was weighed and mixed with water at ratio of 1.76 : 1. Afterwards, 3 aliquots with 45 mg of each prepared from the starch-water premixed product were transferred to DSC pans separately, and used as replicates. The pans were then sealed and placed on the bench at room temperature to equilibrate overnight. A heating rate of 10°C min was used to heat the samples at 30 ~ 130°C. Data was analysed using the instrument software provided by the manufacturer.

### Preparation of granule bound proteins from mature grains

The starch prepared from mature grains by the method described above was used for preparing GBPs of each line. Following the procedures by Rahman et al. (1995) with some modifications, 4 mg of starch was boiled in a protein denaturing extraction buffer (50 mM Tris buffer, pH 6.8, 10% glycerol, 5% SDS, 5% β-mercaptoethanol, and bromophenol blue) at a ratio of 15 μl/mg starch for 5 ~ 10 min. After the centrifugation at 13000 g for 20 min, the supernatant was ready for SDS-PAGE analysis.

### SDS-PAGE and gel staining

Supernatants (25 μl) containing GBPs of each sample together with 5 μl of marker were loaded onto SDS-PAGE gels, respectively. SDS-PAGE was conducted in Nu-PAGE 4-12% gradient gels (Invitrogen) via MOPS-Tris-SDS buffer system containing (50 mM MOPS, 50 mM Tris, 0.1% SDS and 1 mM EDTA) and operated at 20 mA constant current per gel in an Xcell SureLock™ Mini Cell (Invitrogen). After electrophoresis, gels were stained by Sypro Ruby stain (Bio-Rad) following the manufacturer’s instructions, and then visualized under a UV transilluminator (Uvitec, UK). MagicMark™ XP Western Standard protein ladders (Invitrogen) were used to estimate the molecular weight of protein bands.

### Statistical analyses

Statistical analyses were performed using Genstat version 9. Analysis of variance was performed for starch AC, amylopectin structure, RVA and DSC characteristics to obtain the least significant differences at *p* < 0.01, looking at variations among the RILs.
